# “I felt very small and embarrassed by the health care provider when I requested to be tested for syphilis”: barriers and facilitators of regular syphilis and HIV testing among female sex workers in Uganda

**DOI:** 10.1186/s12889-021-12095-8

**Published:** 2021-11-02

**Authors:** Richard Muhindo, Andrew Mujugira, Barbara Castelnuovo, Nelson K. Sewankambo, Rosalind Parkes-Ratanshi, Nazarius Mbona Tumwesigye, Edith Nakku-Joloba, Juliet Kiguli

**Affiliations:** 1 Department of Nursing, College of Health Sciences, Makerere University, Kampala, Uganda; 2 Infectious Diseases Institute, College of Health Sciences, Makerere University, Kampala, Uganda; 3 School of Public Health, College of Health Sciences, Makerere University, Kampala, Uganda; 4 School of Medicine, College of Health Sciences, Makerere University, Kampala, Uganda; 5 Cambridge Institute of Public Health, University of Cambridge, Cambridge, UK

**Keywords:** HIV, Syphilis, Dual testing, Female sex workers, Africa

## Abstract

**Background:**

Periodic testing of female sex workers (FSW) for sexually transmitted infections (STIs) is a core component of global and national responses to achieve population-level STI elimination. We conducted a qualitative study to explore barriers and facilitators of regular syphilis and HIV testing among FSW in Uganda.

**Methods:**

Within a quasi-experimental study among 436 FSW to assess the effect of peer education and text message reminders on uptake of regular STI and HIV testing among FSW, we conducted 48 qualitative interviews in four cities in Uganda from August–December 2018. We purposively selected FSW who tested for syphilis and HIV every 3–6 months; 12 FSW were interviewed in each city. Sex worker interviews explored: 1) reasons for periodic syphilis and HIV testing; 2) barriers and facilitators of testing; 3) experiences of testing; and 4) challenges faced while seeking testing services. Data were analyzed using thematic content analysis.

**Results:**

Thematic analysis revealed individual- and health system-level barriers and facilitators of testing. For syphilis, barriers were a) interpersonal stigma, low perceived severity of syphilis and testing misconceptions (individual); and b) judgmental provider attitudes, paucity of facilities offering syphilis testing, stockouts of test kits and high cost (health system). Facilitators were c) desire to remain healthy, get married and have children, knowing the benefits of early treatment, influence of male partners/clients and normative testing behaviors (individual); and d) sex worker clinics offering dual syphilis/HIV testing (health system). For HIV, barriers included: a) internalized stigma (individual); and b) unfavorable clinic hours, stigma, discrimination, and unfriendly provider (health system). Facilitators were a) motivations to stay healthy and attract clients, habitual testing, self-efficacy, doubts about accuracy of negative test results, and use of post-exposure prophylaxis (individual); and d) availability of testing facilities (health system). Syphilis and HIV had similar testing barriers and facilitators.

**Conclusions:**

HIV programs are likely to be important entry points for syphilis testing among FSW. Multi-level interventions to address testing barriers should consider focusing on these service delivery points. Extending the dual syphilis and HIV testing approach to FSW may improve testing uptake for both infections at public health facilities and decrease population-level incidence.

## Introduction

Periodic testing of female sex workers (FSW) for syphilis and HIV is a pivotal component of global and national responses to achieve elimination of the two diseases at population level [1–3]. Globally, FSW have a 30-times greater risk of HIV acquisition than the general population, and face a disproportionate STI burden [4]. Increasing uptake of HIV and syphilis testing services among FSW features prominently in the global strategy to end the AIDS epidemic by 2030 and the STI epidemic by 2040 [5–8]. The World Health Organization (WHO) recommends that FSW test for syphilis every 3–6 months, and for HIV every 6–12 months [9, 10]. In Uganda, HIV testing services, including targeted moonlight outreach testing campaigns, are widely available at stand-alone voluntary counseling and testing clinics, health centers, hospitals and community outreaches [11–13]. However, despite the high burden of HIV 32–52% [14] and STIs [15] among FSW, regular dual testing for syphilis and HIV is still suboptimal. In 2019, only 14% of the FSW in the four Ugandan cities of Arua, Mbale, Mbarara and Kampala reported testing at least twice for syphilis in the prior 12 months compared with 67% for HIV [16].

Studies have shown that uptake of HIV and other STI testing among FSW is often limited by an interplay of factors operating at individual, societal, structural and policy levels [17, 18]. Factors including poor attitude, low risk perception, fear of positive test results, and low self-efficacy have been reported to limit uptake of HIV and STI testing services at individual level [11, 16–18]. Various studies conducted in India, Zambia, South Africa, Mozambique, and Kenya show that stigma is still a pervasive testing barrier at individual, inter-personal, and societal levels [19–22]. At structural level, long waiting times, lack of privacy and confidentiality, poor physical state of health facilities, discrimination, lack of medicine and test supplies, and uncaring provider attitudes are barriers to testing uptake [11, 18]. Inconvenient clinic hours are a barrier at organizational and policy level [18]. In Uganda, a recent qualitative study found that limited awareness of prevention services, fears, misconceptions, stigma, and inconvenient hours of operation of the clinics were barriers to HIV testing among FSW [11]. Barriers and facilitators of testing may vary for different STIs. However, most of the studies that explore barriers and facilitators of testing in Uganda, and elsewhere on the continent, only focus on HIV.

Scaling up point-of-care (POC) testing for HIV and syphilis increases uptake of testing services by FSW [23] and enables early treatment of both diseases [24, 25]. Routine HIV testing in antenatal care increases testing uptake by FSW [19]. Thus, dual syphilis and HIV testing at HIV clinics could facilitate syphilis screening among FSW and contribute to the WHO global strategy of ending the STI epidemic by 2040 [5, 6]. In this study we explored barriers and facilitators to regular testing for syphilis and HIV among FSW in Uganda.

## Methods

### Study design and setting

This explorative qualitative study was nested in a quasi-experimental study that assessed the effect of peer education and text message reminders on uptake of regular STI and HIV testing among sex workers in Uganda, as previously described [26]. Between August and December 2018, we conducted 48 in-depth interviews with FSW in the four cities of Arua, Kampala, Mbale, and Mbarara (combined population 1,857,088) located in the Northern, Central, Eastern and Western regions of Uganda, respectively [27]. High concentrations of sex work and HIV prevalence have been reported in major cities and towns in Uganda [28, 29].

### Sampling and recruitment

Study eligibility included females aged ≥18 years, self-report of selling sex for goods or money in the prior 12 months, receipt of regular syphilis and HIV testing every 3–6 months, not on antiretroviral treatment (ART) or living with HIV. Potential participants were purposely identified [30, 31] following participation in a questionnaire-based survey to describe regular syphilis and HIV screening practices [16]. Prior to participant recruitment, a mapping exercise was conducted to gain an understanding of time of sex work, sex work hotspots, typologies, sex work procurer connections and territorial management in each of the city, as previously described [16, 26]. Lists of eligible participants describing their demographic characteristics and telephone contacts were drawn for each city. A convenience sample of 12 FSW was interviewed per city. The participants were purposely selected to include FSW from different age and educational strata of 18–24 years, 25–29 years, and ≥ 30 years, and none, primary, secondary, or tertiary, respectively. Prior contact was made with the participants via telephone calls. Participants who declined to participate were replaced with eligible participants on the list within the same city and strata. A total of 48 interviews were conducted.

### Data collection

We conducted a single, in person in-depth interview with each participant. We used an interview guide to explore FSW experiences of regular syphilis and HIV testing, their personal motivations, challenges, and facilitators. Interview guide topics included reasons for periodic syphilis and HIV testing, barriers and facilitators of testing, general testing experiences and challenges faced while seeking testing services. For clarity of interview questions, the guide was piloted with four FSW in a municipality outside the study sites prior to use. Individual interviews were conducted in a language of the participant’s preference (English, Luganda or Runyankore) at venue of their choice where conversations could not be overheard. All interviews were conducted and audio recorded by the principal investigator (RM), after he completed an online research ethics course with emphasis on research with vulnerable populations. All participants provided written informed consent and were reimbursed for their time in accordance with local ethics guidance.

### Data management and analysis

Data were analyzed using thematic analysis, which involves presenting experiences, meanings and realities of participants [32, 33]. All audio recorded interviews were transcribed verbatim and translated into English. Next was the decontextualization or familiarization phase, in which author RM read through all the transcripts to obtain a general sense of information therein and achieve immersion [33, 34]. RM checked all transcripts for errors by simultaneously listening to the audio-recording and proofreading the transcripts. Following decontextualization, RM and two independent qualitative researchers (SJ and WG, PhD trained social scientists not directly involved in data collection) separately prepared the initial codes covering the broader themes (barriers and facilitators) [34, 35]. Codes were compared and synchronized; a codebook was used to code the remaining the transcripts. Emerging codes were condensed into meaningful units [36–38] to generate independent lists of codes, categories, and subcategories. During the process, we constantly compared the data with codes. Codes and sub-categories were contrasted across cities and participant characteristics (age, education, and marital status). The sub-categories and categories were further reviewed by MM, a PhD student with qualitative analysis expertise, and other co-investigators to ensure salient categories were captured and suitable quotes selected for inclusion. The Consolidated Criteria for Reporting Qualitative Studies checklist was used for reporting study findings [39].

### Ethical approval

The study was approved by Makerere University School of Public Health Higher Degrees Research and Ethics Committee, and Uganda National Council for Science and Technology (HS 2403). All participants provided written informed consent in English or their local language. All interviews were conducted at places agreeable to participants where conservations could not be overheard.

## Results

Forty-eight participants were interviewed across the four cities. The median age was 28 years (interquartile range [IQR], 22–29), and the median duration of sex work was 36 months (IQR 24–54) (Table 1). Fifty two percent had obtained secondary education (≥7 years of education) and 31% had primary or no formal education. Half (52%) had never married. FSW solicited clients through bars or clubs (36%), on the street (31%), lodges (18%), and brothels (9%). For two-thirds (63%), sex work was the only source of income. All 48 participants reported testing for syphilis and HIV in the prior three months. Across the four cities, participants reported engaging in risky sexual practices and described their testing practices as habitual.

**Table 1:** Characteristics of participants (*N* = 48)

Variable	N (%) or Median (IQR)
Age (years)	28 (22–29)
Duration of sex work (months)	36 (24–54)
Biological children	2 (1–3)
**Education level**	
Primary or no formal education	15 (31.3)
Secondary	25 (52.0)
Higher education	8 (16.7)
**Marital status**	
Married	3 (6.3)
Separated	13 (27.1)
Widow	7 (14.6)
Never married	25 (52.0)
**Solicitation of clients**	
Street	17 (31.0)
Home	3 (5.5)
Lodge	10 (18.1)
Bar/Club	20 (36.4)
Brothel	5 (9.0)
**Description of sex work**	
Full time, no other source of income	30 (62.5)
Full time supplements income	10 (20.8)
Part-time, have other sources of income	6 (12.5)
Part-time, student	2 (4.2)

### Barriers and facilitators of regular syphilis and HIV testing

**Fig. 1:**
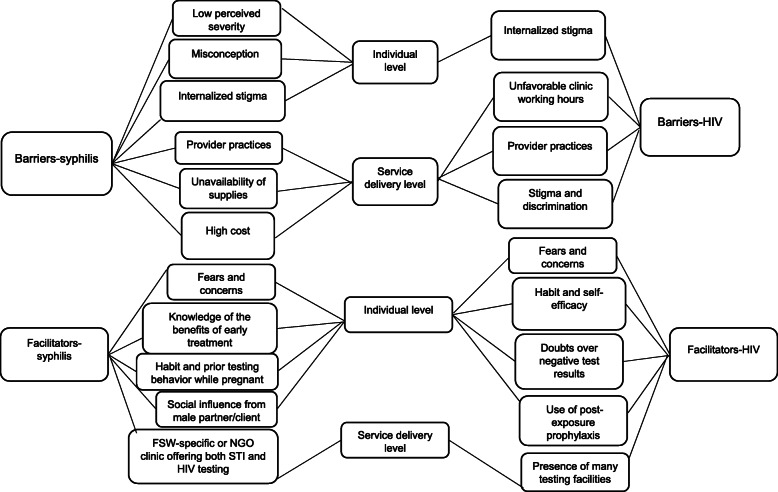
Summary of subcategories and categories of the barriers and facilitators of regular syphilis and HIV testing

Participants described several individual and health system factors as barriers and facilitators of regular syphilis and HIV testing (Fig. 1). Key personal barriers included low perceived severity, misconceptions for syphilis or internalized stigma for both syphilis and HIV. Service delivery barriers included provider practices, stock-outs of syphilis testing kits, high cost of syphilis testing, stigma and discrimination, unfavorable clinic operating hours and uncaring attitudes of HIV providers. Dual testing was mainly facilitated by presence of specific-FSW or non-governmental organization (NGO) clinics that offered integrated STI/HIV testing services. At individual level, testing was mainly facilitated by fears and concerns (i.e., increased HIV risk acquisition, inability to bear children, having syphilis would make one unable to satisfy clients or inability to attract clients, suddenly becoming sick, loss of work and inability to provide for significant others, and psychological anxiety (for HIV) at individual level. A coding tree of the barriers and facilitators of regular of syphilis and HIV testing among FSW is presented in Table 2.

**Table 2:** Coding tree of the potential barriers and facilitators of regular syphilis and HIV testing

Theme	Category	Sub-category	Code	Code frequency
**Barriers**	Low perceived severity		Syphilis is not like HIV, one can live with syphilis, hard to test for it unless sick, syphilis not a big issue, only fear HIV.	32
	Misconception		FSW think you can’t just go and test for syphilis when you have no signs.	33
	Internalized stigma	Fear of being seen at the clinic	I fear finding people I know at the clinic as they could know my work.	6
		Fear of Peers spreading information of one’s status	My friends may see the medical form and spread information if I am positive for syphilis and other STI.	7
	Provider practices	Syndromic STI management	Sometimes you ask for syphilis test, and they simply ask you the signs and write you medicine.	42
		Non-emphasis of syphilis testing	Doctors only encourage us to test for HIV.	30
		Disrespectful and judgmental questioning	I felt small and shamed, the health worker asked me what I was doing when I asked for a syphilis test, I was asked who told me I had syphilis and whether I was a doctor, I was embarrassed	15
		Uncaring attitudes & perceived low quality of care	The care is not good at government hospital, doctors are too busy to attend to us, I spend the whole day at the hospital just to take an HIV test	19
	Unavailability of supplies	Lack of syphilis testing kits	Unless you are pregnant it is not easy to be tested for syphilis at public hospitals, no syphilis testing kits	35
	High cost		At least for HIV you can find many cheap testing services, but testing for syphilis is expensive in private clinics	20
	Stigma and discrimination		We FSW when we visit public hospitals health workers just look at us, once they realize you are a sex worker no one wants to attend to you.	44
	Unfavorable clinic working hours			
**Facilitators**	Fears and concerns	Inability to bear children due to syphilis and other STIs	I test for all STIs because I want to get married, I want to bear children at some point, syphilis may affect your uterus and fail to bear children	7
		Attracting and satisfying clients	In this job you have to be healthy and strong, syphilis and other STI reduces your capacity to satisfy clients, you lose clients.	30
		Worry that syphilis and other STI increase HIV acquisition risk	Syphilis can make you easily get HIV.	25
		Psychological fear of contracting HIV	Start imaging things until at test, I cannot settle until I test, test regularly to get psychological relief.	20
		Suddenly becoming sick and being unable to provide for significant others	Suddenly becoming sick and stop working for my children, I am in this job for my children I have to test every month or 3 months, I can’t allow myself to become sick no one will look after my children.	35
	Social influence	Influence of a regular male partner/boyfriend	Boyfriend insists on STI and HIV testing whenever we meet.	7
		Client demand for unprotected sex	Whenever a client requests for unprotected sex.	4
	Knowledge of the benefits of early treatment		Syphilis heals better if it has not spread widely in the body.	
	Habit and prior testing while pregnant		Now my habit to test every 3 months for HIV, during pregnancy it was a must to test for syphilis and HIV I test for them together, I keep a diary to ensure I test every 3 months for HIV	40
	Self-efficacy		It is easy to test every 3 months for HIV, developed courage to test every 3 months for HIV	44
	Doubts over negative test results		I have messed up a lot but am always told I am HIV negative, I don’t trust test results from outreaches, I doubt a drop of blood from a finger can give true results	10
	Use of post-exposure prophylaxis		To get PEP after unprotected sex, after being forced into live sex to obtain PEP	5
	Presence of specific or NGO clinics offering both STI and HIV testing		I go to specific clinics for FSW that test HIV and other STIs, at Reproductive health Uganda with small money you are tested for HIV, syphilis, and Hepatitis	32
	Availability of many testing facilities		Easy to test for HIV because there are many testing clinics unlike syphilis	48

### Low perceived severity, misconceptions, and internalized stigma

Unlike HIV, syphilis was perceived a less serious health threat by most FSW. They reported that most FSW believed in testing for syphilis after developing the disease. To them this was a barrier to routine testing.

“*Syphilis is not such a big issue like HIV. One can live with syphilis … it is not easy to go and test when you have no signs. I test because when I go at our clinic in Mulago am told to test for syphilis”* (30 year old FSW, Kampala)*.*

Descriptions of internalized stigma as a barrier to both syphilis and HIV testing were amplified by fears of being seen at clinics or peers spreading rumours about one’s perceived status. Rumour mongering was associated with loss of customers and income. These fears were more common among younger participants (≤24 years).

*“I sometimes fear finding people I know at the clinic …*. I *do not want them to know that I am doing this job. Also you are given a medical form if you test for syphilis … your friends may get to know that you have syphilis and other diseases and tell other people which affects your business”* (20 year old FSW, Mbale).

### Provider practices

Syndromic STI management (i.e., treatment without testing), disrespectful and judgemental provider attitudes were reported to limit uptake of routine syphilis testing. Across the study sites, participants reported being prescribed antibiotics for STIs without testing even after requesting to be tested. It was observed that unlike HIV, healthcare workers (HCW) rarely emphasised syphilis testing in public health facilities unless one was pregnant.

*HCWs never emphasize syphilis testing like they do for HIV …. I test for syphilis when I visit the MARPI clinic in Mengo because in other hospitals you are simply asked the signs and symptoms and thereafter they write for you medicines” (*a 34 year old FSW, Kampala).

Among younger participants (≤24 years), it was reported that unlike HIV, requesting a syphilis test was not easy. Such requests were associated with critical questioning from the providers. Participants reported being asked questions like, ‘Who said you have syphilis’? ‘What did you do’? ‘What signs show you have syphilis’? or ‘Are you a doctor in the consultation room’? These questions were perceived as embarrassing, disrespectful, and judgemental.

*“I felt very small and embarrassed by the health care provider when I requested to be tested for syphilis. It is not easy to ask for a syphilis test. Two months ago I requested to be tested for syphilis at the hospital but the doctor looked at me and asked me who told me I had syphilis, what I had done, and which signs …. I felt very small, ashamed and insecure”* (22 year old FSW, Mbarara).

In contrast, uncaring attitudes of HCW were reported to limit uptake of regular HIV testing. Most participants reported that HCW were unfriendly or uncaring especially at public facilities*.* HCW were perceived to be non-responsive and unfriendly to FSW, which led to long waiting times.

“*I went to take an HIV test at government facility but the care was not good … doctors were too busy to attend to us that I spend the whole day in the hospital … .now I test whenever I go to Kampala at a clinic in Mengo”* (30 year old FSW, Mbarara).

### Unavailability of supplies and high cost

Older participants (≥30 years) cited being discouraged to seek syphilis screening due to frequent stock outs of testing kits and medicines. They observed that unlike HIV, testing kits for syphilis and antibiotics were seldom available at public health facilities.

“*Unless you are pregnant, it is not easy to be tested for syphilis in public facilities because testing kits and medicines are not available”* (32 year old FSW, Kampala).

STI testing was reported to be more expensive than HIV testing at private clinics. The low cost of HIV testing was attributed to the availability of several testing clinics.

*“At least for HIV you can find many cheap testing places; testing for syphilis is very expensive in private clinics”* (28 year old FSW in Mbale).

### Discrimination and stigma

Experiences of discrimination were reported as to be a barrier to HIV testing by all participants. However, this mainly occurred at public health facilities unlike sex worker friendly clinics.

*“I used to test for HIV from the main hospital but once HCW realize you are a sex worker no one wants to attend to you or you will be the last to be seen. However, there are many testing canters in Arua …. I now test from Reproductive Health Uganda” (a* 28 year old FSW, Arua).

### Unfavourable operating clinic hours

Some participants reported unfavourable clinic working hours as a barrier to HIV testing. This was mainly in Kampala, the capital city.

*“Like a week ago I went to a city council clinic not far from here. I reached there like at 3:00 pm to take an HIV test … but I was told to come the following day because the clinic had closed … I was very demoralized”* (29 year old FSW, Kampala).

### Fears and concerns

Regular testing for syphilis and HIV was mainly driven by fears and concerns about the impact of syphilis and HIV on one’s own health, work, family responsibilities and future aspirations. Older participants (≥30 years) reported fear of suddenly becoming sick, being unable to work and providing for one’s children as a motivation for their regular testing behaviour. They expressed high risk perception for acquisition of HIV and other STIs, and believed one needed to remain physically attractive and strong to survive in sex work. They reported testing for both HIV and syphilis as they believed poor physical health was associated with failure to attract or satisfy customers. Regular testing meant accessing ART and STI treatment if one was found positive. Testing was also required before getting a post-exposure prophylaxis (PEP) prescription.

“*I fear if I suddenly become sick and stop working … I am in this job for my children. No one will take care of them if I allow myself to become sick. In this job* y*ou have to test for HIV and other diseases such that if found positive you immediately start medicine otherwise you will not be able to* satisfy *customers and continue working* …. *so for me, I test for HIV, syphilis, and hepatitis B at RHU [Reproductive Health Uganda] every 6 months”* (32 year old FSW, Arua).

Younger participants (≤24 years) reported testing frequently for HIV to avoid anxiety. The fear of contracting HIV after condomless sex caused stress, experiences of anxiety, distress, and uneasiness. Condomless sex generates more earnings than sex with a condom and FSW sometimes face a dilemma between needing income and risking HIV infection from unprotected sex. Periodic testing was associated with emotional relief and happiness especially if one tested HIV-negative. The desire for psychological and emotional wellbeing motivated periodic testing for HIV. However, no psychological concerns were reported for syphilis.

*“I sometimes try to insist on condom use but clients offer more money for unprotected sex … .and sometimes I have nothing to eat or feed my child … so I test for HIV almost every month to feel relieved”* (20 year old FSW, Mbale).

Similarly, dual syphilis and HIV testing was reported among participants with childbearing and marriage intentions. Syphilis was believed to cause infertility. The fear of being unable to bear children in the future promoted syphilis testing.

*“In this job one can easily acquire infections that can affect their private parts (vagina) and even the uterus … .for me, at some point, I want to quit this job, get married and have children in future so I normally go to test for syphilis and HIV every two months”* (23 year old FSW, Mbarara).

### Social influences

Some participants reported testing for syphilis and HIV due to the influence of a distant partner or boyfriend who lived in a different part of the country. The partners were usually not aware of their participation in sex work. Their testing behaviour was motivated by request of the partner for fear of being infected.

*“My boyfriend is not aware I do this job … .we usually meet once every four months and normally he insists we must first test for HIV and syphilis”* (23 year old FSW, Kampala).

Sometimes the demand for unprotected sex by male clients influenced frequent HIV testing behaviour. This occurred among younger participants with strong positive body image perceptions or marriage intentions.

*“I test for HIV whenever a client requests for sex without a condom. I am very pretty and many clients want to have sex with me “live” (without a condom) but I only agree if the man agrees and tests negative* (20 year old FSW, Mbale).

### Perceived fidelity of test results

Some participants expressed doubt about the accuracy of their HIV-negative test results. To confirm these results, repeat tests were sought from other clinics. Some FSW reported engaging in unprotected sex, or had HIV-positive clients, but continued to test HIV-negative. Others had doubts about whether a drop of blood drawn from a finger stick could capture their HIV status correctly. When testing was done with a regular client or boyfriend, some FSW sought repeat tests because they feared their client or boyfriend could have bribed clinic staff to change the test result.

*“I do not think a drop of blood from a finger gives correct HIV results … in outreaches, HCW have tested people we know have clients on* antiretroviral *drugs negative …. I have messed up a lot but they continue test me negative … I keep testing because I don’t believe [my test results]”* (32 year old FSW, Kampala).

### Habitual testing and self-efficacy

HIV testing was described as habitual by most participants. Receipt of previous HIV-negative test results gave them courage to test again. Some reported maintaining a diary of testing dates as a reminder.

*“I cannot say no to any man who comes with money. I have made it a habit to test for HIV every three months …. I keep a diary where I record dates*” (28 year old FSW, Arua).

Prior testing for HIV resulted in empowerment and self-efficacy that was frequently described as a facilitator for engaging in regular HIV testing.

“*My parents died of HIV and so my relatives thought I was HIV positive but since I tested negative I have developed the courage to always test for HIV every month”* (26 year old FSW, Kampala).

We observed that some participants tested for syphilis because it was offered at an antenatal clinic.

“*I have learnt to test for syphilis every three months because when I was pregnant it was a must to be tested for HIV and syphilis”* (22 year old FSW, Arua).

### Presence of FSW-specific clinics offering both STI and HIV testing

Participants from Kampala, Mbale and Mbarara cited existence of specific FSW-friendly clinics in Kampala as an enabler of their periodic syphilis and HIV testing behaviours. Reasons for preference of these clinics included testing for all STIs, availability of testing kits and medicines, approachable and caring-healthcare providers among others.

“*Here in Mbarara I went to government facility but the care was not good … now days when I go to Kampala I visit a clinic in Mengo because I can explain my problems to doctors … get counselling and be tested for all STIs … they care about us and I find it convenient*” (28 year old FSW, Mbarara).

NGO clinics that provided integrated services including STI and hepatitis B virus testing at a low cost, promoted syphilis and HIV testing.

“*I usually test at RHU, an NGO clinic because even with small money I am tested for syphilis, HIV and hepatitis B”* (28 year old FSW, Arua).

### Availability of many testing clinics for HIV

HIV testing services were reported to be easily accessible in all cities. All participants cited existence of several testing facilities ranging from free government clinics, NGO clinics, private clinics, and occasionally community-based testing outreaches.

“*For me, it is easy to test for HIV every 3 months because there are many testing centers. I used to test from the main hospital but when healthcare workers know your business (sex work) they take long to serve you … .now I go the health center*” (29 year old FSW, Arua).

These facilities provided choice regarding availability, acceptability, convenience, and affordability. The commonly preferred testing sites were FSW-friendly clinics, public health facilities, and NGO run clinics. Private clinics and community-based outreach campaigns were the least preferred.

*I used to test from a private clinic but I worried a lot waiting for results … .at the main hospital, services are free and healthcare workers prepare you before (pre-test counselling) and after testing … .you get a lot of information about HIV*” (28 year old FSW, Mbale).

Reasons for these preferences included provision of a range of STI testing services, availability of testing kits and antibiotics, approachable and kind HCW for specific FSW-clinics. Public health clinics offered free testing services, health education and pre-test counselling. Private clinics were preferred for being nearby or trusted to provide accurate test results. Outreach campaigns were said to be convenient.

“*I do sometimes test for HIV and syphilis at the MARPI clinic at Mulago especially when I want to get PEP … .but normally I test from private clinics because they are near or when healthcare workers come and test us from here”* (30 year old FSW, Kampala).

## Discussion

Understanding and addressing barriers and facilitators of syphilis and HIV testing among FSW in Uganda is crucial to increasing prevention coverage and decreasing HIV and syphilis burden in this population. Across both individual and health system barriers, our results suggest that much work remains to optimize HIV/STI testing for FSW. This paper shows that FSW face substantial barriers accessing HIV/STI testing because of (1) disrespectful, judgmental, and unfriendly provider attitudes; (2) interpersonal and internalized stigma; (3) unfavorable clinic operating hours; (4) non-emphasis of syphilis testing because of policy recommendations for syndromic STI management; and (5) frequent stock outs of syphilis test kits, high cost of syphilis tests, and low perceived severity of syphilis infection. Given the importance of increasing FSW engagement in HIV/STI care, this study identifies crucial areas that should be addressed at health system and individual level to improve the provider-patient relationship, empower FSW to overcome these barriers to care, and increase utilization of testing services.

Our study shows that stigma was experienced at individual and interpersonal levels. Internalized stigma (negative beliefs someone holds about themselves and their identity) and interpersonal stigma (direct or enacted forms of stigma including judgmental attitudes and having sex workers wait longer for care) are well described barriers to testing uptake because of criminalization of sex work and negative social norms [11, 18, 19, 40]. Internalized stigma was heightened by fears of disclosure of positive HIV or syphilis test results within sex worker peer networks. Reports of discrimination at public health facilities were attributed to interpersonal stigma. FSW commonly experience stigma in health service delivery [41], and negative experiences with HCW are associated with delay or avoidance of testing which is a critical entry point to HIV/STI care [42, 43]. Qualitative work from Kenya and Zimbabwe echoes our finding that stigma from HCW influences health seeking behaviors [43, 44]. Stigma-reduction interventions should include key population sensitivity training for providers, which has been shown to decrease judgemental and discriminatory attitudes among HCW in sub-Saharan Africa [45].

Participants felt it was easier to request for an HIV test at health facilities in contrast to syphilis where such a request attracted embarrassing and critical questioning from providers. This was attributed to the availability of several HIV testing facilities and the normative nature of HIV testing. FSW focused clinics were mainly located in Kampala, whereas public, private, and NGO clinics or community-based outreaches were reported to offer HIV testing in other cities as previously reported [11]. The diversity of HIV testing facilities provided choice about service availability, responsiveness, convenience, and affordability which helped FSW circumvent health system barriers. FSW utilization of health services was also limited by unfavorable clinic operating hours, unavailability of test kits, and high cost, factors previously reported as structural barriers to HIV testing uptake [11, 17, 20]. Specialized FSW and NGO clinics were preferred because they offered integrated STI and HIV services, shorter waiting times, availability of treatment, friendly and caring HCW. Similar findings have been reported from India, Mozambique, Kenya and South Africa [20, 46]. However, most of these studies focused on HIV testing.

Our finding that regular testing was motivated by fears and concerns over maintaining health, ability to work, family responsibilities, and future aspirations or social influences, and was facilitated by testing habits and self-efficacy is consistent with prior work in which perceived benefits of testing or attitudes, self-efficacy, social influences and past behavior have been reported to influence STI testing [16, 47–49]. STI risk perception was high, but disease severity was perceived differently. HIV was perceived a serious health threat and testing meant getting psychological respite or access to treatment to extend one’s life or prevent HIV, work to provide for self and significant others. In contrast, syphilis was perceived a less serious threat and the misconception that one needed to develop symptoms before testing was widespread as previously reported [50]. Syphilis testing was facilitated by dual syphilis/HIV testing which is standard-of-care during pregnancy in Uganda [51], and was motivated by concerns over satisfaction of customers, HIV acquisition risk, and infertility. The belief that syphilis increased risk of HIV acquisition or reduced capacity to satisfy customers was common among older participants. Younger participants with childbearing and marriage intentions believed syphilis caused infertility. Some of these beliefs and misconceptions show lack of comprehensive knowledge about syphilis. Despite the paucity of data on drivers of syphilis testing among FSW, our findings support evidence showing that knowledge of testing benefits and attitudes influences STI testing behaviors [16, 17, 52]. At individual level, fear of being seen at the clinic was a barrier to dual syphilis and HIV testing.

Social influences (i.e., influence of referent others) [53] motivated testing among participants with distant regular partners. Male partners were usually unaware of female engagement in sex work and HIV testing occurred whenever they met or in anticipation of meeting because the partner demanded pre-sex testing for fear of being infected. Fear of infecting a male client has been reported to motivate HIV testing among FSW in Russia [52]. However, in our study fear of being infected by male partners and clients following payment of larger sums of money for unprotected sex motivated periodic HIV testing among FSW with high self-efficacy. Empowerment interventions including skills training that have been found to build the self-efficacy and promote HIV testing among FSW [18] should be strengthened, and tailored to the specific needs of FSW. Surprisingly, doubts over the accuracy of HIV-negative test results was reported as a motivation for periodic testing despite also being documented as a barrier [11]. This was mainly due to beliefs that a FSW cannot test HIV-negative because of sexual risk behaviors or that a drop of blood from finger stick cannot give correct test results. Such sentiments indicate lack of comprehensive knowledge about HIV that should be strengthened in HIV educational campaigns to FSW. The desire to confirm test results, get psychological satisfaction or access treatment prompted testing frequently at various health facilities.

Public facilities were reported to have limited focus on syphilis serological screening despite offering HIV pre-testing counselling, health education, and free services. In this setting, public health programs emphasize HIV testing for FSW [11–13, 54] perhaps because of funding priorities or testing guidelines. Additionally, syndromic STI management is standard-of-care in this setting. This may explain why providers rarely encouraged syphilis testing. Our findings suggest programs should also emphasize periodic testing for syphilis and other STIs. Uganda guidelines now recommend dual syphilis and HIV for pregnant women and their partners [51]. Dual testing was introduced to achieve elimination of mother-to-child transmission of HIV and syphilis. Extending such services to FSW could change attitudes of health providers, including messaging on routine syphilis testing, and improve testing uptake for the two infections. Higher uptake of syphilis screening services among FSW is a key pillar of the 2040 global efforts to end the STI epidemic [5, 55].

The clinical and policy implications of our findings include assessing fears and concerns about regular syphilis and HIV testing at service delivery points, tailoring health promotion messages to addressing these fears and concerns, developing messages emphasizing routine syphilis and HIV testing in HIV and ANC clinics for FSW, developing and strengthening stigma-reduction interventions for FSW and health providers, and extending the dual syphilis and HIV testing approach to FSW.

The strengths of our study include a qualitative exploration of barriers and facilitators of dual syphilis and HIV testing among a geographically diverse sample of FSW in Uganda. Our findings contribute to the evidence base of HIV and STI testing in a setting where several studies focus only on HIV. The limitations of this explorative qualitative study include purposeful sampling of FSW who reported engaging in regular syphilis and HIV testing behaviors. Hence our findings may largely reflect FSW who are knowledgeable about benefits of regular testing and have self-efficacy despite encountering barriers to testing. We only used in-depth interviews to collect data. However, focus groups are not a good choice for in-depth study of sensitive topics and limit interaction with individual participants. Additionally, we did not explore the influence of brothel owners and pimps on testing behaviors of FSW. Nevertheless, similar findings have been reported in studies employing in-depth interviews, focus group discussions, and key informant interviews.

## Conclusions

This study shows that despite the existence of multilevel barriers, regular HIV testing among FSW is facilitated by availability of testing facilities and welcoming healthcare environments. Availability of syphilis serological testing services was very limited. Interventions to improve uptake of syphilis and HIV testing among FSW in Uganda need multilevel frameworks targeting both FSW and providers and policy makers. Community empowerment programs that address knowledge gaps and misconceptions should emphasize periodic testing for both syphilis and HIV. To increase availability of testing services, there is need to recommend dual syphilis and HIV testing for FSW in national guidelines, provide sensitization training for HCW and deliver services while respecting human rights of FSW.

## Data Availability

The datasets used during the current study are available from the corresponding author on request. The questionnaire is included as supplementary information.

## Abbreviations

FSW: female sex workers
HIV: human immunodeficiency virus
STI: sexually transmitted infection
WHO: World Health Organization
